# Very Early Short‐Course Cerebrolysin After Tenecteplase and Mechanical Thrombectomy: A Case Report

**DOI:** 10.1155/crnm/9962315

**Published:** 2026-08-28

**Authors:** Hanna Ziąbska, Adam Kobayashi, Michał Karliński

**Affiliations:** ^1^ Department of Neurology and Neurodegenerative Diseases, Institute of Psychiatry and Neurology, Warsaw, Poland, ipin.edu.pl; ^2^ Department of Neurology, Stroke and Vascular Diseases, Department of Pharmacology and Clinical Pharmacology, Institute of Medical Sciences, Cardinal Stefan Wyszynski University, Warsaw, Poland, uksw.edu.pl

**Keywords:** cerebrolysin, functional recovery, intravenous thrombolysis, mechanical thrombectomy, stroke, tenecteplase

## Abstract

Despite high recanalization rates with mechanical thrombectomy (MT), large‐vessel occlusion stroke results in death or dependency in over half of patients. Cerebrolysin, a multimodal neuropeptide preparation, may attenuate ischemia–reperfusion injury and support early neurorehabilitation. We report a 88‐year‐old woman with moderate prestroke disability who presented 2 h after the onset of right hemiparesis and aphasia due to M2 occlusion of the left middle cerebral artery. She received intravenous tenecteplase followed by MT, achieving complete reperfusion after three passes with no direct complications and no immediate clinical improvement. Directly postprocedure, cerebrolysin 30 mL/day was initiated as adjunctive treatment. Over the next 24 h, neurological deficits gradually resolved with no hemorrhagic transformation or evident infarct on follow‐up CT. As the patient did not require early intensive rehabilitation and regained her prestroke functional status, cerebrolysin was discontinued after three doses. This report is not intended to establish the therapeutic efficacy of short‐course adjunctive cerebrolysin. It illustrates how clinical signals from randomized and observational studies can be interpreted for the purpose of individual therapeutic decisions.

## 1. Introduction

Stroke remains a leading cause of death and disability worldwide, even with contemporary workflows in well‐developed systems with good access to reperfusion therapies [1, 2]. In large‐vessel occlusion (LVO) strokes, mechanical thrombectomy (MT) achieves successful recanalization (mTICI 2b–3) in over 80% of patients, but only about 38% regain functional independence [2]. These reflect the complex pathophysiology of ischemia‐reperfusion injury, including rapid growth of the ischemic core prior to recanalization, microvascular “no‐reflow,” distal microembolization, and disruption of the blood–brain barrier (BBB) [3]. Recent STAIR recommendations have therefore emphasized that future cerebroprotective strategies should be tested as add‐ons to timely reperfusion rather than as stand‐alone treatments, targeting both neuronal survival and microcirculatory integrity [4]. In parallel, multiple randomized trials and meta‐analyses have established tenecteplase as the recommended alternative to alteplase in patients eligible for intravenous thrombolysis (IVT), especially in the 4.5‐h time window or considered for MT [5].

We report an elderly patient with moderate prestroke disability treated for acute M2 occlusion with tenecteplase‐assisted MT followed by a very early short course of cerebrolysin. While no inference regarding efficacy can be drawn from a single observation,

Our case illustrates how clinical signals from randomized and observational studies can be interpreted for the purpose of individual therapeutic decisions.

### 1.1. Case

An 88‐year‐old woman was transported by ambulance to the Neurological Emergency Department at 11:57, two hours after sudden‐onset right‐sided limb paresis and aphasia. Her medical history included untreated hypertension, osteoporosis, and chronic low back pain limiting ambulation (modified Rankin Scale [mRS] score of 3).

**FIGURE 1:**
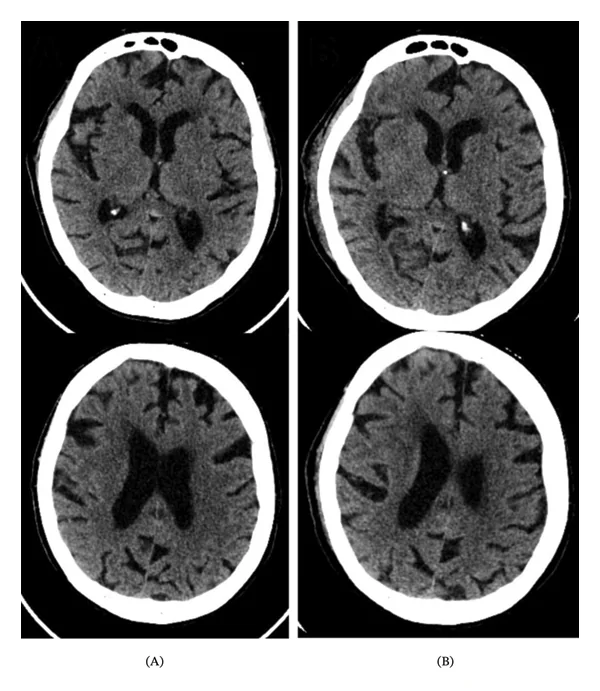
Noncontrast CT on admission (A) and 24 h after reperfusion therapy (B). No hemorrhagic transformation or visible infarction is seen on follow‐up CT.

On admission, she scored 10 points on the National Institutes of Health Stroke Scale (NIHSS): questions and single‐step commands (2 + 1), right‐sided limb paresis (1 + 1), severe mixed aphasia (2), and right facial nerve palsy with dysarthria (2 + 1). Noncontrast brain computed tomography (CT) at 12:20 showed a hyperdense sign in the left middle cerebral artery (MCA) with no clear early ischemic changes (Alberta Stroke Programme Early CT score of 10, Figure 1). Subsequent CT angiography confirmed the M2 occlusion of a left MCA branch. At that point, the patient was deemed potentially eligible for both IVT and MT.

**FIGURE 2:**
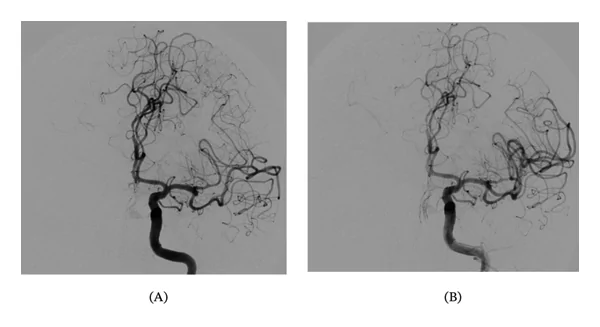
Digital subtraction angiography before thrombectomy (A) and after recanalization (B) of the middle cerebral artery M2 branch.

After transfer to a monitored bed (blood pressure 133/70 mmHg and heart rate 80/min) and a point‐of‐care blood glucose check (134 mg/dL), at 12:36, she received 20 mg of tenecteplase (0.25 mg/kg). The endovascular procedure was started under general anesthesia at 13:15. After three passes (two aspirations with the RED62 catheter, then one combined aspiration thrombectomy with the RED62 catheter and the Solitaire X 3 × 20 mm stent retriever) at 13:44, she achieved complete reperfusion (mTICI 3) with no immediate clinical effect (Figure 2). After return from the angio suite, Cerebrolysin 30 mL intravenously was administered as adjunctive treatment on the basis of a biologically plausible cerebroprotective rationale and available clinical safety data [6, 7]. This decision was made after the procedure and was not part of a predefined protocol. No direct procedural complications or adverse events temporally associated with cerebrolysin were observed.

We observed gradual neurological improvement reaching NIHSS 0 by the next morning. Cerebrolysin was discontinued after the third daily dose because the patient had complete neurological recovery, no hemorrhagic transformation on 24‐h CT, and no stroke‐related deficit requiring intensive early neurorehabilitation (Figure 1).

After a comprehensive diagnostic workup, she was diagnosed with an embolic stroke of undetermined source. The only abnormal laboratory finding was an elevated LDL cholesterol level (150 mg/dL). The patient was discharged home on acetylsalicylic acid and high‐dose atorvastatin with no neurological deficits (NIHSS 0, mRS 3). At the 3‐month follow‐up, her condition remained stable (NIHSS 0, mRS 3).

## 2. Discussion

This case report illustrates the safety, feasibility, and potential usefulness of using cerebrolysin as additional therapy in combination with IVT and MT.

Cerebrolysin is an intravenously administered mixture of low‐molecular‐weight peptides and amino acids derived from porcine brain tissue with multimodal actions, including antiexcitotoxic, antioxidative, and anti‐inflammatory effect, and promotion of neuroplasticity and synaptogenesis [8]. Cerebrolysin modulates receptors involved in glutamatergic, GABAergic, and cholinergic signaling, corresponding to protective effects against excitotoxicity and apoptosis. Its anti‐inflammatory effects is supposed to counteract the destabilization of BBB [7]. Both randomized and nonrandomized studies did not reveal any important safety issues [3, 6, 7, 9–16]. A meta‐analysis of 9 randomized trials by Bornstein et al. reported that cerebrolysin initiated within 72 h of stroke onset was associated with better 90‐day functional outcomes in patients with moderate‐to‐severe stroke, without excess serious adverse events [9]. In contrast, the more recent Cochrane meta‐analysis was neutral for cerebrolysin and raised concerns about imprecision and risk of bias [10]. This apparent discrepancy stems from methodological differences, namely, imposing the 48 h therapeutic window, that resulted in the exclusion of the positive CARS trials [11]. The 2021 European Academy of Neurology guidelines provide a weak recommendation for its use for at least 10 days as part of early motor rehabilitation after ischemic stroke, especially if NIHSS is ≥ 8 [17]. This concept has been reinforced by the ESCAS trial on poststroke aphasia and in the recently published CREG‐2 registry that included patients with baseline NIHSS 8–15 [12, 13].

More directly relevant to our case report, the nonrandomized CEREC‐Stroke study showed that patients with baseline NIHSS  ≥ 8 and futile recanalization who received 14–21 days of cerebrolysin had lower 12‐month mortality (13% vs. 43%) and numerically fewer hemorrhagic transformations (HT, 8% vs 33%) compared with controls [14]. In the randomized CEREHETIS trial, early addition of cerebrolysin to alteplase significantly reduced the rate of symptomatic intracranial hemorrhage (sICH, 3% vs 9%), but with no clear effect on 90‐day functional outcome [15]. A more recent prospective, nonrandomized, blinded‐endpoint study (*N* = 100) focusing on patients undergoing successful MT for anterior‐circulation LVO reported that add‐on Cerebrolysin initiated ≤ 8 from the onset and continued for 21 days resulted in significantly lower occurrence of HT (14% vs. 40%) and sICH (2% vs. 24%), with a higher proportion of good functional outcome at 90 days (68% vs. 44%) [3]. A similar propensity score matched analysis in cardioembolic LVO also showed lower rates of HT (20% vs 57%) and sICH (3% vs 41%), with a higher proportion of good outcomes (64% vs 35%) and even lower mortality (5% vs 33%) [16]. Taken together, previous studies provide a biologically and clinically plausible rationale for combining cerebrolysin with reperfusion therapy to attenuate reperfusion injury and potentially improve the translation of radiological success into clinical recovery [6, 7].

Our patient differs in several important respects from the populations studied in these trials. Firstly, she represents a clinically relevant but largely underrepresented in trials group of very elderly patients with prestroke dependency not related to neurological deficits. Second, her cerebrolysin regimen was initiated immediately after MT and discontinued after three daily doses, unlike the regular 10–21‐day courses used in most studies. Such a schedule may be better suited to targeting early BBB stabilization and reperfusion injury than to longer‐term neurorestorative effects. However, direct measures of BBB stabilization were not available in this case.

It is important to note that our patient had several well‐established predictors of complete neurological recovery, including early presentation, absence of early ischemic changes on baseline CT, an M2 branch, intravenous tenecteplase before thrombectomy, and complete angiographic reperfusion. Previous controlled studies indicate that very early adjunctive cerebrolysin could have contributed to getting the full potential of recanalization without hemorrhagic transformation [6, 7]. The patient required three passes to achieve mTICI 3, and her neurological improvement was gradual rather than immediate. Nonetheless, this case cannot determine to what extent cerebrolysin contributed to the clinical or radiological outcome, and further randomized studies are warranted.

## 3. Conclusion and Potential Treatment Strategy

Based on available evidence from randomized trials and observational studies, very early initiation of cerebrolysin (30 mL/day i.v.) may be considered as an adjunct therapy to IVT with tenecteplase or alteplase and/or MT in selected cases [3, 6, 7, 14–16]. The intended rationale is twofold:

▪ To increase the direct benefit from reperfusion therapy by mitigating ischemia–reperfusion injury and reducing the risk of hemorrhagic transformation;
▪ To support early recovery and facilitate timely rehabilitation when indicated.

This approach may be most relevant in moderate ischemic stroke (NIHSS 8–15), particularly in patients with cortical deficits, including upper‐limb weakness or nonfluent aphasia [7, 9, 11–13]. If clinically significant deficits persist after reperfusion and intensive rehabilitation is required, cerebrolysin may be continued for a conventional 10–21‐day course, as suggested by EAN 2021 rehabilitation guidelines [17].

In patients with rapid and near‐complete clinical recovery, it seems reasonable to discontinue very early initiated cerebrolysin after 2‐3 doses. The present case illustrates the feasibility of such a treatment strategy. However, new data from academic‐driven randomized trials are needed to guide decisions in everyday clinical practice.

## Funding

No funding was received for this manuscript.

## Consent

According to the journal policy, written informed consent for publication was not required because the report contains fully deidentified clinical and imaging data and no directly identifying information. No names, initials, date of birth, hospital number, patient’s image, or other direct identifiers are included.

## Conflicts of Interest

The authors declare no conflicts of interest.

## Data Availability

The data that support the findings of this study are available from the corresponding authors upon reasonable request.
